# Public Opinion on European Health Policy, Lessons from the COVID-19 Pandemic

**DOI:** 10.3390/ijerph19084813

**Published:** 2022-04-15

**Authors:** Maria Denisa Vasilescu, Simona Andreea Apostu, Eva Militaru, Eglantina Hysa

**Affiliations:** 1 The Faculty of Economic Cybernetics, Statistics and Informatics, The Bucharest University of Economic Studies, 15-17 Dorobanti Street, 010552 Bucharest, Romania; maria.vasilescu@csie.ase.ro; 2 National Scientific Research Institute for Labor and Social Protection, 6-8 Povernei Street, 010643 Bucharest, Romania; militaru@incsmps.ro; 3 Institute of National Economy-Romanian Academy, 050711 Bucharest, Romania; 4 Department of Economics, The Faculty of Economic and Administrative Sciences, Epoka University, 1032 Tirana, Albania; ehysa@epoka.edu.al

**Keywords:** health policy, European Union, COVID-19 pandemic, vulnerable groups, logistic regression

## Abstract

Often, global crises, such as the COVID-19 pandemic, bring to light crucial weaknesses in political, economic, social and health systems. First, there are governments who formulate and implement policies and, second, there are the citizens who support them, thus contributing a great deal to their success. Our paper investigates the European citizens’ opinion on health policy, focusing on their preference for European health policy during the coronavirus pandemic. The paper uses bibliometric analysis, descriptive statistics, and logistic regression to discuss the public opinion on health policy, the factors of influence, the change in perspectives between 2020 and 2021, and the socio-demographic profile of those favorable for the development of a European health policy in response to the coronavirus pandemic. Our findings show that citizens from southern and central European countries are more likely to prioritize the development of a European health policy, as compared to Nordic countries. Between 2020 and 2021, pro-European health policy citizens profile changes and becomes clearer, from pensioners to young working age males with medium education. In general, people prioritizing a European health policy value health as the most important issue at a national level are generally satisfied with the European Union and do not trust their national government.

## 1. Introduction

On 11 March 2020, the World Health Organization (WHO) declared the spread of coronavirus as a pandemic [1]. The pandemic had multiple effects worldwide, starting from millions of infected people, high death numbers, and crisis in healthcare systems, which was followed by threat to the economy [2,3]. Furthermore, due to the ongoing coronavirus pandemic, some crucial global weaknesses have been identified in our health and political systems to respond to emergent challenges effectively and efficiently [4]. From one side, there is the government’s role as a policy-making body that formulates and implements strategies to achieve the proper goal-driven outcomes that target at best the society’s needs and prosperity [5,6,7]. Often the governments are experiencing a lack of fundamental factors that lead to a successful organization, for instance, a clear statement of purpose, goals, values, agreed statements of organizational interactional rules, etc. [8]. On the other side, there is the capacities and effectiveness of the health sector to best respond to emergencies and crises of enormous increased demand toward the need for this sector. However, it is essential to have continued investment in R&D, infrastructure, and human resources that are related to the health sector in order to foster sustained development [9] and be prepared for such emergencies. Moreover, Sturmberg et al. [4] in their study emphasized the need for a robust distributed health system and for transparent communication as the basis for trust in the system. According to this study, investing in the health sector would help in the redesign of strong health systems that could respond to the current and future health crises, and as such, over time we expect to have healthier, more resilient, and highly productive societies, striving toward sustained development.

However, to the best of our knowledge, there is a lack of review articles that synthesize the opinion on health policy in Europe in time of the coronavirus crisis. It is important to find out what the people perceive and think because this information would address the right inquiries to the public policymakers and raise their awareness on the next targets and goals to be achieved. Understanding public opinion, especially its dynamics, is of crucial interest and very helpful in order to track the political effects, as it moves from the debate over its passage to its implementation and operation [10]. Indeed, it is important to have empirical analysis exploring public opinions that reflect the leadership role of the governments, policies applied, response to emergencies, and lessons learned during crisis times.

Therefore, this paper aimed to identify the opinion on health policies in Europe regarding the COVID-19 pandemic, and the main components associated with it, with a dynamic analysis being possible using data from two Eurobarometer surveys from 2020 and 2021. The paper used bibliometric analysis, descriptive statistics, and logistic regression analysis to address the following research questions:

RQ1. What is the opinion on health policy in Europe during the pandemic?

RQ2. What are the factors of influence in the response to the pandemic?

RQ3. What are the perceptions of Europeans in 2021, in the midst of the pandemic, compared to 2020, when it started? What lessons did we learn?

RQ4. What is the profile of a person in favour of developing a European health policy in response to the coronavirus pandemic?

These research questions are vital to identify the public opinion related to health policy in order to create an appropriate framework to implement and operate this policy by governments. Identifying the population’s position and profile regarding the health policy and the factors influencing the perception of health policy at the beginning and in the midst of the pandemic also highlighted the opinion dynamics, the pandemic’s impact on this perception and resistance and resilience to a health crisis.

Furthermore, this study contributes to the literature on health policies and responsibility aspects by also including some related factors to the emergency cases such as a pandemic. The empirical results indicated that this pandemic conduced to increasing the awareness related to health policy and its implications and may serve as crucial information for further research and discussion, as well as to address better policies in the future.

Thus, our paper is organized as follows: the second section offers an overview of the main findings of the literature in terms of health policy development, the third part of the paper presents the data and methodology applied to verify the research hypotheses, the fourth section describes the results of the empirical analysis, and the final section reflects the conclusions.

## 2. Literature Review

The development of health policy is highlighted through response in terms of crisis [11]. Thus, the health crisis indicates the shortages and the measures needed to be implemented in order to face and resist.

In case of the severe acute respiratory syndrome (SARS) outbreak in 2003, the response was the creation of the European Centre for Disease Prevention and Control (ECDC) and a mechanism for joint procurement of vaccines [12].

The coronavirus disease outbreak in 2019 became the most urgent public health issue threatening lives all over the world [13]. The health crisis generated by COVID-19 was confronted with limited EU governance frameworks, widely criticised [14,15], with the response indicating low resilience and resistance [16].

The response to the pandemic in order to minimize the viral transmission aimed to reduce the incidence of face-to-face contact [17]. The policies in the case of the coronavirus pandemic represented the biggest concern of public health in the last two years, requiring professional advocacy attempts through appropriate inter-sectoral collaboration and government coalitions [18].

The impact of this pandemic was huge, creating changes in global communication and technical advances in modelling [19]. Political leaders developed solutions for easing lockdowns based on effective reproduction data [20,21,22,23,24], leading to public awareness regarding modelling infectious disease. The emergencies and lockdowns imposed by the healthcare regulators and governments led to an adverse effect on the mental health of the people [25].

The response to the COVID-19 pandemic required coordinated efforts as in disaster management [26]. Solutions consisting of containment and mitigation efforts were aimed at saving lives, thus avoiding human capital losses and flattening the pandemic curve. However, all these measures reduced economic activity [27,28], transforming the health crisis into an economic crisis, too. Thus, this pandemic became both a widespread global pandemic and an economic disaster [29].

The coronavirus crisis is still present in our lives, not knowing its trend in the future, and the repercussions on the population will be felt for years from now [30]. The measures imposed by governments to fight the pandemic, and the fear of illness, led to anxiety and mental health degradation amongst people [25].

Although it has been shown in the literature [31,32,33,34,35,36] that the population did not know how to face emerging infectious diseases, there are also studies [37,38,39] according to which good knowledge, attitude and practice lead to strengthening the community and residents’ health education [40].

**Figure 1:**
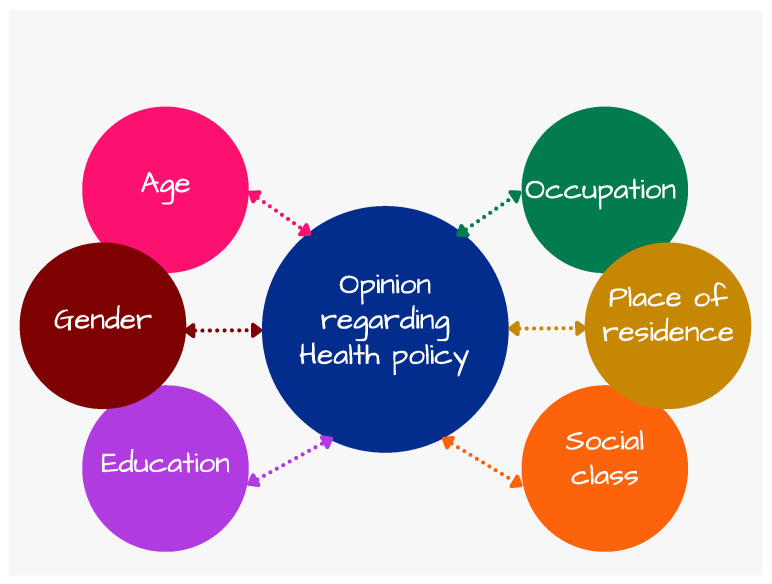
An overview of the main factors affecting health policy. Source: Authors’ design.

Population opinion on health policy development is influenced by several factors, both demographic and socio-economic. An overview of the main factors affecting the opinion regarding health policy is represented in Figure 1.

To predetermine and shape health systems, outcomes, and policy, a crucial factor is represented by gender [41]. Gender equity represents a vital factor to influence the quality of public healthcare systems and their outcomes [42]. Women are more cautious [43], risk-averse [44,45], fatalistic [46], and loss averse [12]. There are also differences between gender in risk-taking behaviour in the case of leaders [47]. Reporting on a group, male leaders assume high risks, while female leaders assume small risks [48].

The COVID-19 pandemic also registered gender differences, affecting more men than women [49]. The explanation consists of a lower immune response in the case of men and practices and behaviours related to masculinity, such as smoking and drinking, engaging less in preventive public health measures and delayed healthcare seeking [50,51]. Men present a higher prevalence of comorbidities, such as cardiovascular disease, diabetes, and hypertension, conducive to severe COVID-19 [52].

Another major factor influencing the opinion on public health is represented by the human environment, consisting of population density, urbanization, and age structure [47]. Higher population density is associated with active transport, more perceived stress, and smoking, leading to high mortality [53] and poor health conditions [54]. Population significantly influences both socio-economic and health system policies. As the immune system is inversely correlated with age, affecting the physical strength of elderly to respond to infection, the relationship between physical health condition and age is also negative [55,56].

Social class is another factor influencing health policy as lower social classes are associated with less education, with people registering less control over their external environments [57]. Social class is also correlated with individuals’ beliefs [58]. One indicator reflecting social class is income, with empirical studies indicating that higher income reduces beliefs in conspiracy theories [59], the relationship between conspiracy beliefs and income being indeterminate [60].

Occupation is associated with health policy, and people working in finance, for the government or in the military exhibit the lowest levels of conspiracy thinking [61]. Occupations are associated with wages [62] in order to compensate the increase in risk [63], therefore affecting health policy development.

Although urban and rural areas share common concerns [64], place of residence significantly influences and contributes to health policy [65], residents in rural areas being less willing to pay for the health insurance and less involved in what concerns health policy development [66]. Place of residence also implies geographic differences concerning dietary preferences, affecting health policy.

Other factors influencing the opinion on health policy are: trust concerning health policy [30], satisfaction with implemented health policy [67,68], and the health crisis impact over their income and lives [69].

As people’s major concerns were about education and healthcare policies [70], citizens’ satisfaction with government policies was also associated with economic performance [71,72,73,74] and trust [75,76,77,78,79,80], increased satisfaction among people leading to higher trust in government [81].

For measuring people’s attitudes towards public policies, other important factors are: political ideology [82], income [83], and employment status [30].

In order to create a comprehensive image of the literature regarding the opinion on health policy in Europe in the time of the coronavirus crisis, we used bibliometric analysis. We investigated all published papers provided by the Web of Science platform related to the association of the words: “COVID”, “Europe”, “health”, “policy”. The result is represented by 1458 articles from 2020 until March 2022.

Bibliometric analysis provides quantitative results of the literature [84] in order to determine emerging trends, to examine the intellectual structure of a specific domain [85,86,87] and to provide retrospectives of journals in milestone years [88]. Its main advantage is deciphering and mapping the scientific knowledge by structuring large volumes of data in rigorous ways [89].

The bibliometric methodology comprises the application of quantitative techniques on bibliometric data, such as publications or citations [90,91], with the main aim to extract and manipulate data [92]. This analysis involves the identification of the literature content within a given subject area, the results being of major importance to policymakers, scientists or other stakeholders [84]. Thus, bibliometric analysis is considered a state-of-the-art methodology, including components from all scientific domains [93].

Quantitative measures are also provided by content analysis through harvesting of keywords [94,95]. This method discovers up-and-coming fields, the extracted data highlighting substances that are unknown by the population [96,97].

As shown in Figure 1, the monthly average of published papers and the number of citations in the area illustrates a hyperbolical progression jump in 2022 regarding the number of publications and in 2021 regarding the number of citations. Thus, there has been a growing interest in the field in the last two years, with the main focus being on health policy development.

Exploring the amount of information offered by the word clouds, we identified the most common words found in the scientific articles. The co-occurrence of authors’ words of the publications are investigated, taking into account a frequency of at least 20 times, using a correlation degree greater than 0.5 and a threshold of 0.5. The analysis was realized using Vos programme.

**Figure 2:**
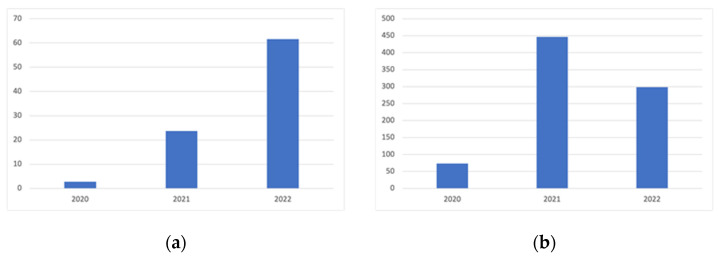
Dynamics on (**a**) publications, and (**b**) citations in the field. Source: Authors’ selection from WoS database, based on selected words, using Excel.

In order to recognize the most common words, we used cluster analysis on a keyword network, which was extracted from the papers. The results are presented in Figure 2, highlighting the words that record the highest frequencies of occurrence: “study”, “pandemic”, “country”, “research”, “innovation”, “data”, “population”, “outbreak”, “government”, “age”, “sex”, “inequality”, “social care”, “treatment”.

**Figure 3:**
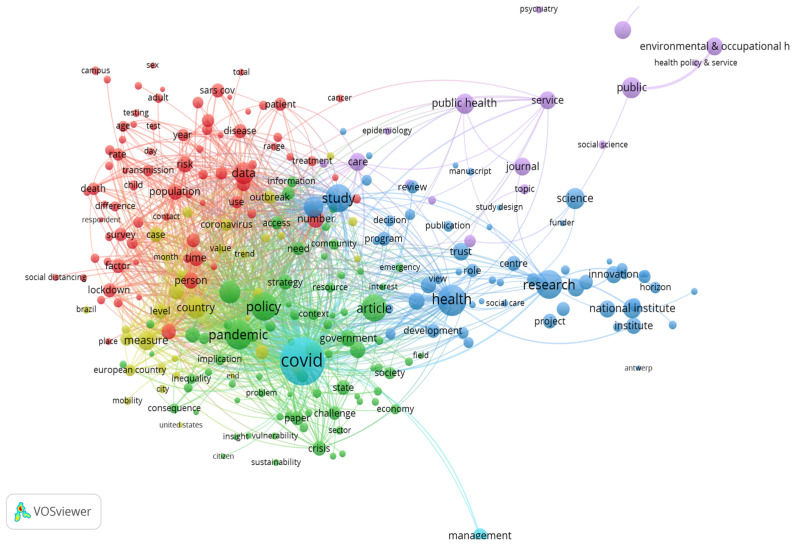
Most common words and word network in scientific publications’ content. Source: Authors’ selection from WoS database, based on selected words, using VOS programme.

The combinations of most encountered words were explored by the most correlated words within the selection of articles. The empirical results (Figure 3) highlighted six significant clusters of the most common combinations in the selected 1458 studies in the field. These are:

- Cluster 1: addition, adult, burden, association, campus, cancer, child, concern, condition, cost, death, diagnosis, disease, difference, effect, evaluation, factor, gender, hospital, incidence, infection, intervention, lockdown, mental health, mortality, quality, public health policy, prevalence, restriction, risk, social distancing, symptom, treatment, transmission, vaccination.
- Cluster 2: access, action, activity, attitude, benefit, capacity, challenge, change, community, crisis, demand, economy, effort, emergency, employment, environment, evidence, experience, focus, future, government, health system, healthcare, impact, implementation, impact, income, importance, inequality, information, interest, knowledge, lack, opportunity, recommendation, response, relationship, sustainability, uncertainty, vulnerability, strategy.
- Cluster 3: collaboration, commission, control, decision, development, drug, economic, education, financial support, health research, health policy, horizon, innovation, medical research, ministry, national, national institute, partnership, practice, role, preparation.
- Cluster 4: account, coronavirus, effectiveness, epidemic, end, measure, mobility, outbreak, policy maker, prevention, spread, world, world health organization.
- Cluster 5: care, communication, epidemiology, ethic, frontier, general and internal, infectious disease, medical informatics, public health, social science, technology, topic, medicine, journal.
- Cluster 6: COVID, green, management.

As can be observed from the cluster composition, the first cluster is associated with health dimension, the second cluster reflects the factors of influence, and clusters 3–6 consider measures in order to fight the virus, from healthcare to the economic sector.

## 3. Data and Methodology

The data used in this research were collected through Eurobarometer 94.3 conducted between February and March 2021, and Eurobarometer 93.1, for which the data were collected between July and August 2020. The data source is GESIS-Leibniz Institute for Social Sciences. Eurobarometer 93.1 addressed issues such as the COVID-19 pandemic, European priorities and citizenship, the European Union budget, as well as general opinions and attitudes towards the EU; it includes 33,059 units and 544 variables. Eurobarometer 94.3 focused on similar topics, including pandemic issues, EU priorities, general opinions and attitudes towards the EU, media use and political information; it includes 38,718 units and 479 variables.

Both Eurobarometers use the individual as statistical unit, the sampling procedure is probabilistic, stratified, and as methods of data collection, computer-assisted face-to-face interviews (CAPI/CAMI) and web-based self-administered questionnaires were used (CAWI). The company that collected the data is Kantar, the surveys being requested by the European Commission.

For our study, the variable of interest was the opinion of the respondents regarding the development of a health policy at European level, as a priority measure to respond to the coronavirus pandemic. The analysis of this variable was carried out comparatively, during the two years affected by the pandemic, to capture possible changes in European citizens’ attitudes as they get used to the fact that the pandemic is part of everyone’s long-term life (the perceptions in 2021) and not just a short-term shock, as it was initially perceived (in 2020). This variable, as well as the other variables used in the quantitative analysis, is described in Table 1.

To have a preliminary understanding of the data used, we have presented in Table 2 some essential information of descriptive statistics, such as the number of observations, the mean, and the standard deviation.

To achieve our research objective, we chose to use logistic regression, because it allows investigating the factors that influence respondents’ opinions on a European health policy, as well as identifying the profile of the individual with increased chances to consider developing such a policy a priority.

In the logistic regression model, the dependent variable is binary, being coded with 1 in case of success and with 0 in case of failure [98]. In this case, the conditional mean of the regression model is:

(1) E(y|X)=1·P(y=1|X)+0·P(y=0|X)=P(y=1|X) 

The probability described above can only take values between 0 and 1, so we cannot use any linear regression function, but only one that respects the condition of values between 0 and 1 and therefore a model response:

(2) $$p=P\left(y=1|X\right)=f\left(\alpha +\beta_{1}x_{1}+\beta_{2}x_{2}+\ldots +\beta_{k}x_{k}\right),\;\mathrm{with}\;p\in \left[0;1\right].\;$$

The logistic regression model makes a connection between the explanatory variables and probabilities, as follows:

(3) $$p=f\left(\alpha +\beta_{1}x_{1}+\beta_{2}x_{2}+\ldots +\beta_{k}x_{k}\right)=\frac{\exp\left(\alpha +\beta_{1}x_{1}+\beta_{2}x_{2}+\ldots +\beta_{k}x_{k}\right)}{1+\exp\left(\alpha +\beta_{1}x_{1}+\beta_{2}x_{2}+\ldots +\beta_{k}x_{k}\right)}.\;$$

It can be easily verified that this function takes values only in the range 0 to 1. It also results that:

(4) $$log\frac{p}{1-p}=\alpha +\beta_{1}x_{1}+\beta_{2}x_{2}+\ldots +\beta_{k}x_{k}$$

The value *p*/(1 − *p*) links the probability of success (*p*) with the probability of failure (1 − *p*) and is called the odds of success. The value *log* [*p*/(1 − *p*)] is the logit of p and represents the log odds of success. Thus, the logistic regression model becomes a linear function for the odds of success that uses the logit transformation to model a binary response variable as a linear function of the explanatory variables.

Regarding the interpretation of the logistic regression model coefficients, if all other variables are constant, a one-unit change in the explanatory variable *x*_1_ leads to a change in the log odds of success by $\beta_{1\;}$. units. This implies that the odds of success change by a multiplicative factor$\;exp\left(\beta_{1}\right)$, called the odds ratio.

## 4. Results

The first step in our study was a descriptive statistical analysis of EU citizens’ perceptions on prioritizing the development of a European health policy and on how their opinions changed in early 2021, when the pandemic had already become the “new normality”, compared to the summer of 2020, when people where still struggling to adapt to a life with restrictions.

**Figure 4:**
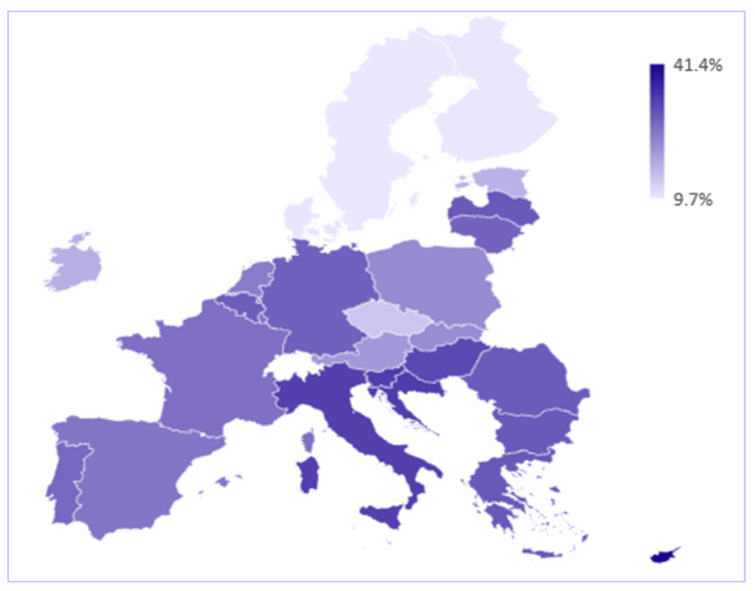
Share of individuals agreeing with the development of a European health policy, 2021. Source: Authors’ calculation using SPSS and Excel.

The distribution, by country, of people prioritising the development of a European health policy in 2021 is presented in Figure 4. Cyprus stands out with the highest registered value, 41.4% of the respondents being in favour of a European health policy in response to the coronavirus pandemic. Next, we noticed a group of countries with high shares of individuals prioritising the European health policy among the measures to combat the health crisis: Slovenia and Croatia with 33.3%, Italy with 32.8% and Hungary with 31.6%. At the other end of the hierarchy, we find the Nordic countries, Sweden, Finland, and Denmark, with a maximum share of 10% of individuals who agree that the development of a health policy at the European level should be prioritised as a measure to fight the pandemic.

**Figure 5:**
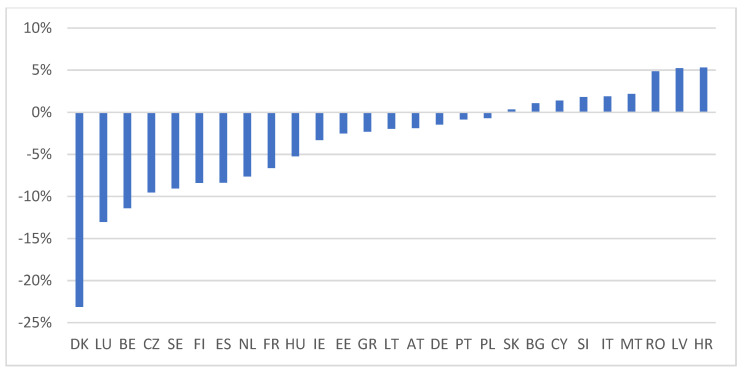
The growth rate of the share of people who prioritize the development of a European health policy, 2021 compared to 2020. Source: Authors’ calculation using SPSS and Excel.

Regarding the change in perceptions between the two moments of time that we analysed, Figure 5 shows that in most states the share of people who agree that the development of a European health policy is a priority in the fight against the pandemic has decreased. A particularly sharp decline is recorded in Denmark, of about 22 percentage points. Thus, the perception of the Danes changed dramatically in just a few months. At the opposite pole are countries such as Croatia, Latvia, Romania, Malta, Italy, Slovenia, Cyprus, and Bulgaria, the only member states for which there has been an increase in the share of people eager for health policy at the European level.

We can highlight the situation in Cyprus: both in 2020 and in 2021 it was in the top positions, with values well above the European average. This result may be explained by the need for closer cooperation with other EU members, in the sense that there have been many bottlenecks in the health system during the pandemic, both in terms of supply chains and the occupancy rate of hospital beds, and a well-coordinated policy at European level could have been helpful.

At the same time, although at first sight, the downward trend in the appetite for a European health policy seems bizarre, a possible explanation could be the panic that characterized the beginning of the pandemic, when countries were struggling to cope with the dramatic health and economic crisis. Over time, the panic has diminished as both the authorities and the people have been able to better manage the coronavirus pandemic.

The analysis continued with the construction of a logistic regression model in which the dependent variable represents the opinion of the European Union’s citizens regarding the development of a health policy at the European level, with the value 1 indicating the agreement of individuals that such a policy is a priority in the fight against the coronavirus pandemic. This approach has made it possible to analyse in more detail, at the individual level, the demographic characteristics as well as the contextual factors that influence the opinions regarding European health policy.

For the explanatory variables included in the model, described in the previous section, the correlation matrix was calculated in order to avoid multicollinearity. Based on the results obtained, we decided to eliminate from the analysis two variables strongly correlated with the others, namely the indicator that measures satisfaction with the COVID-19 measures taken by local authorities and the variable that quantifies the confidence in the measures taken at the EU level to fight the coronavirus pandemic.

Thus, the general form of the estimated logistic regression model was:

(5) $$european\_\mathrm{health}\_policy=\beta_{0}+\beta_{1}\times age+\beta_{2}\times gender+\beta_{3}\times education+\beta_{4}\times employment\_status+\;\beta_{5}\times residency+\beta_{6}\times social\_class+\beta_{7}\times fin\_consequences+\beta_{8}\times health\_problem+\beta_{9}\times measures\_nat\;+\beta_{10}\times measures\_EU+\beta_{11}\times trust\_health\_auth+\beta_{12}\times trust\_nat+\beta_{13}\times trust\_EU+\varepsilon_{t}$$

We performed the Hosmer–Lemeshow goodness of fit test for both logistic regression models, with the results indicating that our models adequately fit the data. A relatively common problem when using logistic regression on small samples is estimation bias, but this is not the case for the regression models we have developed, because the samples we used are very large (19,486 observations in 2020 and 21,002 observations in 2021).

The results of the logistic regression for both years are presented in Table 3. In 2020, very few statistically significant variables were obtained. Of the socio-demographic characteristics considered, only employment status significantly influences the respondents’ perception regarding the development of a European health policy, the pensioners being the ones who are more prone to have this opinion, compared to employed persons. For the other variables included in the study, the results are generally in line with our expectations. People who consider health to be the main problem nowadays are 1.3 times more likely to prefer the development of a European public policy. Additionally, those who generally trust the European Union are more likely to agree with a European health policy as a priority to manage the pandemic.

The model for 2021 highlighted that people in the 25 to 39 years age group are more likely to agree to a European health policy compared to young people. Gender has been shown to be a statistically significant variable, with men being more prone to wanting a European health policy. The level of education also plays an important role in shaping the opinions of the respondents, those with medium education being 1.2 times more likely to prefer a European health policy compared to low-educated individuals. Regarding employment status, the results indicated that retired people are more likely to consider that a European health policy would be beneficial, as compared to employed persons. The type of community is not statistically significant, indicating that there are no important differences in opinions between rural and urban residents in this matter. However, the social class of the respondent has an impact, as people who consider themselves to belong to the upper-middle or higher class of society are less likely than those in the middle class to prioritise a European health policy as the main response to the coronavirus pandemic. Additionally, individuals severely affected financially by the pandemic are 1.3 times more likely to prefer the EU’s integrated health policy than those who have not had such difficulties. Thus, we can identify a greater predilection of the financially vulnerable to consider that a European health policy should be a priority in the fight against the pandemic.

The analysis of the contextual variables included in the study showed a higher chance of prioritizing a European health policy among those who consider health to be the most important issue at the national level, among those satisfied with anti-COVID measures taken at the EU level, and among those who are generally satisfied with the European Union. Moreover, it is worth noting that respondents who generally do not trust the national government tend to prefer the development of European health policy as a key measure in the management of the coronavirus pandemic.

The comparative analysis of the logistic regression models for the two years highlights first of all that the results are quite different. The model for 2021 is better and contains more statistically significant variables. Thus, a first observation could be that in 2020 a certain profile of the individual who values the development of a European health policy is not precisely outlined, as there are no significant differences in the sociodemographic characteristics.

The results obtained for 2021 allowed us to draw the profile of a person who is in favour of developing a European health policy as the main response to the coronavirus pandemic: They are more likely to be a man, aged between 25 and 39 years, with an average level of education, who does not belong to the upper-middle or higher class of the society and who has been severely affected financially due to the pandemic. Additionally, the individual portrayed is more likely to consider health a major issue at the national level, to have general confidence in the European Union, and to be satisfied with the measures taken during the pandemic by the EU, while he tends not to trust the national government.

## 5. Conclusions

The pandemic caused by the coronavirus has affected the whole world, both in terms of health and socially and economically through the restrictions imposed. This crisis has highlighted the health systems’ stats and the resistance and resilience in facing a pandemic. Thus, the leaders realized the need for adequate health policies and the need to develop existing ones.

In this context, our paper analyses the opinions of citizens towards health policy in Europe, in 2020 and at the beginning of 2021, in order to capture the pandemic effects. Another fact pursued in our work is the characterization of citizens who consider the development of health policy a priority.

The analysis results showed that the largest share of citizens prioritizing the development of a European health policy is found in Cyprus, Croatia, Slovenia, Italy and Hungary. The countries with the lowest share of citizens who prioritize the development of health policies are: The Nordic countries, Sweden, Finland, and Denmark.

In 2021, in most countries, the share of people who consider the development of health policies at the European level a priority has decreased; in Denmark there was a decrease of 22 percentage points. Instead, Croatia, Latvia, Romania, Malta, Italy, Slovenia, Cyprus and Bulgaria, have registered an increase in the share of people eager for health policy at the European level.

The profile of the citizen who admits that it is necessary to improve the health policy, in 2020 is: pensioners, people who consider health to be the main problem nowadays and those who generally trust the European Union.

In 2021, the citizen profile has changed, including people aged between 25 and 39, men, medium education, retired people, middle class, and those severely affected financially by the pandemic. The type of community is not statistically significant, registering no differences between rural and urban residents in this matter.

Additionally, the people prioritizing a European health policy are people considering health to be the most important issue at the national level, those satisfied with anti-COVID measures taken at the EU level, and those who are generally satisfied with the European Union. People who generally do not trust the national government tend to prefer the development of European health policy as a key measure in the management of the pandemic Coronavirus.

Although the two periods analysed are quite close, the difference regarding the opinion of the citizens is big. If for the year 2020 the profile of the individual who values the development of European health policy is not precisely outlined, in 2021 it was well defined, probably due to the multiple changes that occurred during this period.

This study contributes to the literature on health policies and responsibility aspects by including some related factors to emergency cases such as a pandemic. Due to the pandemic affecting all activities, this paper also has a significant contribution to industry and policymakers.

The pandemic has highlighted that the healthcare system needs crucial improvements and the areas where the most are required in order to successfully face a health crisis in the future. Furthermore, it also illustrated the importance of both reacting rapidly and considering specific sociocultural aspects in the context of an epidemic [99,100]. Thus, adequate health policies are absolutely necessary, and for this, one of the most important issues is awareness from both the population and governments.

The results of this study highlighted an increased awareness related to health policy in 2021 in the case of the European population, and this may serve as crucial information for further research and discussion as well as to address better policies in the future. The public opinion during COVID is also different from the opinions under normal conditions, with one of the lessons of this pandemic being the awareness regarding the importance of the health system and of the public policies in force. In this context, our manuscript specifically contributes to the literature on health policies and responsibility, with the empirical results indicating that this pandemic led to increasing the awareness on health policy and its implications.

This study has limitations, some due to reliance on survey data and some due to the lack of relevant working papers. Regarding the relevant literature, there are not many significant papers related to our theme since we considered the public opinion in the time of the coronavirus pandemic. The limitations related to the survey data are consisting of limiting the data availability, with it being necessary to consider the questions in the survey.

Future research directions will focus on how public opinions might be affected by financial factors, both at the individual and the national level. Individual investment decisions may play an important role in the presence of health expenditure risks under the threat of COVID-19 [101], with public opinions being biased in the presence of COVID-19-induced sovereign credit risk [102].

## Figures and Tables

**Table 1:** The description of variables.

Variable	Description
Age	Age of the respondent, a categorial variable with four groups: 15 to 24 years, 25 to 39 years, 40 to 54 years, and 55 years or older.
Gender	Gender of the respondent, a binary variable with value 1 for men and 0 for women.
Education	Level of education, a categorial variable with three levels: low (up to 15 years of education), medium (between 16 and 19 years of schooling) and high (20 years of education or more).
Employment status	Employment status of the respondent, a categorial variable with three variants: employed (includes self-employed persons), retired, and unemployed or house persons.
Type of community	Place of residency, a binary variable with value 1 for urban and 0 for rural areas.
Social class	Social class of the respondent, a categorial variable with three levels: lower or working class, middle class and upper middle or higher class. This variable is used as a proxy for income or standard of living.
Financial consequences	Serious financial consequences due to the pandemic, a binary variable with value 1 indicating that the respondent was severely affected by the pandemic financially. The variable is used as a proxy for economically vulnerable individuals, due to pandemic.
Satisfaction COVID measures—local	A binary variable with value 1 indicating that the respondent is very satisfied or fairly satisfied with the measures taken by the local authorities to fight the coronavirus pandemic.
Satisfaction COVID measures—national	A binary variable with value 1 indicating that the respondent is very satisfied or fairly satisfied with the measures taken by the national government to fight the coronavirus pandemic.
Satisfaction COVID measures—EU	A binary variable with value 1 indicating that the respondent is very satisfied or fairly satisfied with the measures taken by the European Union to fight the coronavirus pandemic.
Health an important issue	A binary variable with value 1 indicating that the respondent considers health to be the most important issue at national level at the time of the interview.
Trust in health authorities	A binary variable with value 1 indicating that the respondent generally tends to trust the health authorities and medical staff in its country.
Trust in national government	A binary variable with value 1 indicating that the respondent generally tends to trust the national government.
Trust in EU	A binary variable with value 1 indicating that the respondent generally tends to trust the European Union.
Trust EU for pandemic	A binary variable with value 1 indicating that the respondent totally trusts or tends to trust that EU will make the right decisions in the future, considering its response to the coronavirus pandemic.
European health policy	A binary variable with value 1 indicating that the respondent agrees or totally agrees that the development of a European health policy should be a priority in the response to the coronavirus pandemic.

**Table 2:** Summary statistics.

Variable	2020			2021		
	N	Mean	Std. Deviation	N	Mean	Std. Deviation
European health policy	26,678	0.280	0.449	27,409	0.245	0.430
Age	33,055	3.018	1.019	38,699	2.964	1.012
Gender	33,055	0.464	0.499	38,718	0.484	0.500
Education	29,840	2.252	0.684	32,815	2.377	0.664
Employment status	30,818	1.434	0.627	38,718	1.764	1.036
Type of community	33,052	0.669	0.471	38,707	0.675	0.468
Social class	32,707	1.588	0.648	38,366	1.597	0.681
Financial consequences	31,442	0.547	0.498	38,367	0.506	0.500
Health an important issue	32,549	0.322	0.467	38,717	0.414	0.493
Satisfaction COVID measures—national	32,188	0.671	0.470	38,425	0.508	0.500
Satisfaction COVID measures—EU	29,712	0.544	0.498	36,824	0.517	0.500
Trust in health authorities	27,315	0.799	0.401	26,997	0.806	0.396
Trust in national government	26,775	0.446	0.497	37,616	0.428	0.495
Trust in EU	29,956	0.506	0.500	36,739	0.548	0.498
Valid N (listwise)	18,161			21,002		

**Table 3:** The results of the logistic regression.

Explanatory Variables		MODEL 1-Year 2020		MODEL 2-Year 2021	
		B	Exp (B)	B	Exp (B)
Constant		−1.175 *	0.309	−1.653 *	0.192
Age	Age (15–24 years)-ref				
	Age (25–39 years)	−0.014	0.986	0.215 **	1.240
	Age (40–54 years)	0.038	1.038	0.139	1.149
	Age (55 years and older)	0.059	1.061	0.169	1.184
Gender (1 = man)		0.038	1.039	0.070 **	1.073
Education	Low (up to 15 years)-ref				
	Medium (16–19 years)	0.083	1.087	0.149 **	1.161
	High (20 years or more)	0.045	1.046	−0.019	0.981
Employment status	Employed-ref				
	Retired	0.131 *	1.140	0.123 *	1.131
	Unemployed or house persons	0.024	1.024	−0.055	0.946
Type of community (1 = urban)		−0.065	0.937	0.058	1.060
Social class	Middle class-ref				
	Lower or working class	−0.036	0.965	0.000	1.000
	Upper middle or higher class	−0.085	0.918	−0.210 *	0.810
Serios financial consequences due to pandemics		0.026	1.027	0.226 *	1.254
Health-the most important issue		0.251 *	1.285	0.217 *	1.242
Satisfaction COVID measures-national		0.046 **	1.047	−0.052	0.949
Satisfaction COVID measures-EU		−0.057 *	0.944	0.118 *	1.125
Trust-health authorities		0.043	1.044	−0.048	0.953
Trust-national government		0.004	1.004	−0.159 *	0.853
Trust-EU		0.123 *	1.131	0.192 *	1.212
Number of individuals included in the analysis		19,486		21,002	
Nagelkerke R Square		0.09		0.17	

Source: Authors’ calculation in SPSS using Eurobarometer data. * 1%, ** 5% significance level.
